# Storing, combining and analysing turkey experimental data in the Big Data era

**DOI:** 10.1017/S175173112000155X

**Published:** 2020-11

**Authors:** D. Schokker, I. N. Athanasiadis, B. Visser, R. F. Veerkamp, C. Kamphuis

**Affiliations:** 1 Animal Breeding and Genomics Centre, Wageningen University & Research, Droevendaalsesteeg 1, P.O. Box 338, Wageningen 6700 AH, The Netherlands; 2 Laboratory of Geo-information Science and Remote Sensing, Wageningen University & Research, Droevendaalsesteeg 3, P.O. Box 47, Wageningen 6700 AA, The Netherlands; 3 Hendrix Genetics Research, Technology & Services B.V., Spoorstraat 69, P.O. Box 114, Boxmeer 5831 CK, The Netherlands

**Keywords:** data lake, sensors, extract, transform and load, scalability, machine learning

## Abstract

With the increasing availability of large amounts of data in the livestock domain, we face the challenge to store, combine and analyse these data efficiently. With this study, we explored the use of a data lake for storing and analysing data to improve scalability and interoperability. Data originated from a 2-day animal experiment in which the gait score of approximately 200 turkeys was determined through visual inspection by an expert. Additionally, inertial measurement units (**IMUs**), a 3D-video camera and a force plate (**FP**) were installed to explore the effectiveness of these sensors in automating the visual gait scoring. We deployed a data lake using the IMU and FP data of a single day of that animal experiment. This encompasses data from 84 turkeys for which we preprocessed by performing an ‘extract, transform and load’ (**ETL**-) procedure. To test scalability of the ETL-procedure, we simulated increasing volumes of the available data from this animal experiment and computed the ‘wall time’ (elapsed real time) for converting FP data into comma-separated files and storing these files. With a simulated data set of 30 000 turkeys, the wall time reduced from 1 h to less than 15 min, when 12 cores were used compared to 1 core. This demonstrated the ETL-procedure to be scalable. Subsequently, a machine learning (**ML**) pipeline was developed to test the potential of a data lake to automatically distinguish between two classses, that is, very bad gait scores *v*. other scores. In conclusion, we have set up a dedicated customized data lake, loaded data and developed a prediction model via the creation of an ML pipeline. A data lake appears to be a useful tool to face the challenge of storing, combining and analysing increasing volumes of data of varying nature in an effective manner.

## Implications

Our work showcases the use of a data lake, a tool from the domain of Information Technology, in the animal sciences domain. A data lake can handle ever-increasing volumes of data, varying data structures as well as data from different sources. Data from an animal experiment investigating locomotion of turkeys were stored and analysed within a dedicated data lake. We demonstrated the data lake scalability and potential for easy data analyses. This will become of more importance when in the (near) future larger experiments will be performed or as sensor technology will be used for real-time decision-making.

## Introduction

Traditionally, animal scientists work with comprehensive data acquired from animal experiments to perform their research. Such data sets are collected through carefully designed experiments to test a predefined hypothesis, resulting into manageble volumes of high-quality data. However, with the rise of the Internet of Things, the expectation is that sensor technologies will be used on a larger scale in the near future, resulting in much higher volumes of data than traditionally used. Sensor technologies are increasingly being adopted to monitor, for example, animal health and welfare in cattle (Smith *et al.*, 2006; Matthews *et al.*, 2016), pigs (Guarino *et al.*, 2017) and poultry (Nääs *et al.*, 2010). Ongoing technological innovations and their implementation within the animal sciences domain increase the generation of both unstructured non-relational data (i.e. camera or video images) and structured data that are not standardized (i.e. Internet of Things sensor recordings). This transition is leading animal science into the era of Big Data, where data sets are of lower fidelity and are collected fast, in high volumes, and where equipment may vary significantly in less coordinated ways. With this increasing availability of large amounts of data of varying nature, there is a new challenge raises: how to store, combine and analyse these animal data efficiently.

In the Information Technology (**IT**) domain, there is a transition from structured relational databases to schema-less databases (i.e. the NoSQL databases) for storing unstructured data and multimedia, and from data warehouses to data lakes to avoid extensive upfront data modelling. A data lake is a ‘centralized repository containing virtually inexhaustible amounts of raw (or minimally curated) data that is readily made available anytime to anyone authorized to perform analytical activities’ (Terrizzano *et al.*, 2015). The key driver behind these transitions in the IT domain is the need to handle ever-increasing data sets, with varying data structures and formats (multimodality), as well as data from different sources (heterogeneity) and originating from various data channels which may be subjective or objective. Moreover, it is not known *a priori* for what purpose data will be used or the purpose may change over time. The re-purposing of data has been further amplified by recent developments in data analytics like the large amounts of data being used in machine learning (**ML**). These changes make ineffective the classic data integration paradigm, where a global data schema can be built with modest effort and subsequently all data sources can be mapped to (Golshan *et al.*, 2017). Instead, data lakes allow for managing data in an ‘pay-as-you-go’ way (Golshan *et al.*, 2017). In a data lake, data are stored in their original format and are applied *only when needed* through an extract, transform and load (**ETL**-) procedure that extracts data out from their original format, transforms them into a desired usable format and loads them to make them available for further processing.

The objective of this study was to investigate how a data lake be utilized for storing and analysing large volumes of data in the animal sciences. We used data collected during a gait score experiment in turkeys, where inertial measurement units (**IMUs**), a force plate (**FP**) and a 3D-camera were used to capture (indices) of the gait scores. The original output of these sensors was stored in a data lake and subsequently an ETL-procedure was performed. We showcase the scalability of the ETL-procedure in a data lake by simulating increasing volumes of data up to 30 000 turkeys and report the wall time for preprocessing them. Furthermore, we have generated an ML pipeline where we employed a random forest to classify two groups of turkeys, that is, those with a normal or an abnormal gait score.

## Material and methods

### Data collected during the animal experiment

Within the Breed4Food (a public-private partnership) ‘Locomotion’ project, an animal experiment was set up to collect gait scoring information from turkeys (Visser *et al.*, 2018). A gait score is traditionally assigned to each turkey during a so-called tomwalk. During such a tomwalk, turkeys walk one by one through a small alley where their gait is scored by an expert. This gait score, running from 1 to 6, is subsequently used to select turkeys for breeding; turkeys with a low score will be removed from the flock, whereas turkeys with higher scores are kept in the breeding programme. To study whether this visual scoring can be automated, this animal experiment also used three sensors: three IMUs (Xsens MTw Awinda, Xsens Technologies B.V, Enschede, The Netherlands) were attached on each turkey, one was attached on the neck and one on each leg. Moreover, a FP (Kistler, Kistler B.V. Nederland, Eemnes, The Netherlands) was placed in the alley of the tomwalk, and a 3D-camera (Intel Realsense D415, Intel, Santa Clara, CA, USA) was placed at the start of the alley, filming the turkeys as they walked through the alley from behind. Staff recorded the time and turkey identification each time a turkey started the tomwalk, and when time allowed these metadata were already ‘cleaned’, by changing the folder name to the animalID for which the data were recorded. When this was not the case, the sensor and animalID were linked by starting or stopping time of the recordings. The gait scoring was performed for two consecutive days, resulting in sensor data from 84 turkeys on day 1 and 100 turkeys on day 2. In this study, we only focus on the first day of the animal experiment, because changes in the experimental set-up were made after the first day. All turkeys received a gait score by visual inspection of the same trained expert. Table 1 displays the summary statistics of the collected data, including sensor data from the FP and IMU, as well as the catergorized gait scores.

**Table 1:** Summary statistics for the sensory data and the expert assigned gait score of turkeys

Sensor	Attribute	Unit	Type	Minimum	Maximum	Mean	SD
Force plate	Walk duration	Seconds	Numeric	0.69	14.7	2.5	1.9
Force plate	Max vertical axis force	Newton	Numeric	13.75	57.27	19.5	5.6
Inertial measurement units	Roll axis sign changes	Dimensionless (integer)	Numeric	3	2568	406.2	445.3
				*Class distribution*			
Human expert	Gait score	Dimensionless (ordinal)	Categorical (two classes)	Very bad (30), otherwise (54)			

### Constructing a dedicated customized data lake

A data lake typically involves Big Data technologies such as the Hadoop Distributed File System (**HDFS**) for storage and Apache Spark (Zaharia *et al.*, 2016) for deploying the ETL-procedure and analytic pipelines, over a computer cluster. In this study, we developed a virtual machine for deploying Apache Spark and HDFS locally, and conneting to a remote cluster on Amazon Web Services, using Flintrock. The virtual machine was developed for the specific animal experiment at hand, as a Docker container. The Docker container includes all the required software for data collected during the animal experiment: Python, R and Scala support for Apache Spark, along with Jupyter Notebook for creating interactive notebooks and PixieDust for interactive visualization. It also includes all customized scripts in Python and C++ that are required for extracting our animal experiment data from their original raw format. All developed scripts, Jupyter notebooks and the Docker container are available online through github, https://github.com/BigDataWUR/locomotiondatalake.git, using an open-source license.

### Testing the extract, transform and load procedure

The usefullness of a data lake for storing, combining and analysing data effectively requires two major steps: the ETL-procedure and the data analyses.

**Figure 1:**
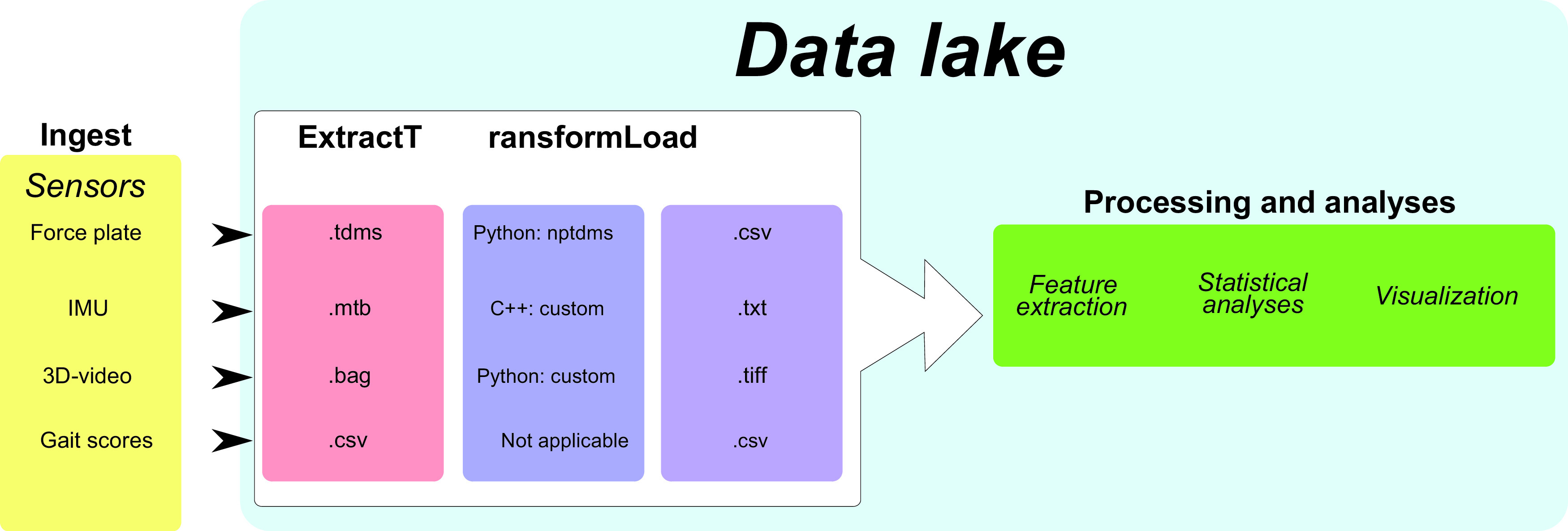
Flow diagram of the data lake. Turkey data are first ingested into the data lake, followed by the ‘extract, transform and load’ procedure, and lastly the data can be processed and analysed. IMU, inertial measurement unit.

The ETL-procedure involves extracting raw sensor data, transforming them into a desired format and loading them to make them available for processing and analyses (in step 2). This ETL-procedure is depicted graphically in Figure 1. In our case study, both the FP and the IMUs generated binary data (.tdms and .mtb, respectively), and customized scripts had to be developed to transform these data into formats useful for analyses further downstream (Figure 1). The extracted binary FP data were transformed into comma-separated values (**CSV**) files via the cross-platform Python package npTDMS, which reads and writes TDMS files as produced by LabVIEW (https://nptdms.readthedocs.io/en/latest/). For the extraction and transformation of the binary IMU data, a C++ script was written to automatically extract the information and convert these into a CSV file. The 3D-video images were stored in bag files, a file format in Robot Operating Systems for storing a sequence of records. These were extracted by using a custom-made Python script that links to the Robotics Operating System (version *Melodic Meriona*).

At the end of the ETL-procedure, all raw sensor files were transformed to an open data format and were linked to animal identifiers before making them available for downstream processing and analyses.

To test the scalability of a data lake, and demonstrate its capacity to process large volumes of data, we artificially generated data sets of different sizes. First, a subset of the original data set has been selected, containing the original data from three turkeys. This encompasses the FP, IMU, (3D) video data, as well as the BWs and gait scores. This subset has been made available as an open-access turkey data set on Zenodo (https://doi.org/10.5281/zenodo.3563513). Then, by duplication we generated 4 data sets of 30, 300, 3000 and 30 000 turkeys. Subsequently, we deployed an Apache Spark cluster of one master node and six worker nodes of two cores each on Amazon Elastic Cloud, using Flintrock, which is a command-line tool for launching Apache Spark clusters. All cluster nodes were configured as Elastic Cloud ‘m3.large’ instances, with 2 vCPU, 7.5 GB RAM and 32 GB of instance storage hard disk. Apache Spark version 2.3.3 was installed along with Hadoop HDFS version 2.7.

We used our customized script and executed this on a single machine, and used the required computation time as a reference value. Subsequently, we executed the same script, now using Apache Spark on the deployed cluster, where we used a six different cluster sizes (2, 4, 6, 8, 10 and 12 cores). This resulted in a total of 24 experiments (4 data sets that differ in size multiplied by 6 different cluster sizes). In each of these experiments, we measured the wall time to turn the .tdms files into .csv. CSV to test the ability of a data lake to work with larger volumes of data.

### Testing the scalability of data analyses

After transforming the binary data into open CSV formats, the second step involves analyses of the data. To explore how this is performed in a data lake, we build a data analytics pipeline. This pipeline included the generation of features from the sensor data in the lake, linking them with the visual gait scores based on animal identification, initializing a classification model and tune parameters of this model to identify the best performing model. In our experiment, this data analytics pipeline loaded involved data from the FP and IMU recordings from day 1 (84 turkeys). From the FP sensor, the pipeline extracted two features: (1) the duration of the turkey being on the forceplate and (2) the highest recorded force on the vertical axis. From the IMUs, the pipeline extracted the number of sign changes in the y-axis of the ‘roll’ parameter. It is important to realize that these features may not have any biological meaning. They were easy features to extract and are selected just to demonstrate how the data analytics pipeline works. The next step in the pipeline was to employ a random forest algorithm to use these features for a binary classification of the turkeys on whether they will be used for breeding or not (i.e. gait score 1 *v*. gait score >1). As the used data set was relatively small with just 84 turkeys, we evaluated the random forest using a 5-fold cross-validation, also using Apache Spark’s ML package. Tuning the parameters of the random forst was done using the area under the receiver operating curve (**AUROC**) and the area under the precision-recall curve (**AUPRC**), which are commonly used in binary classification problems (Davis and Goadrich, 2006). Tuning parameters involved (1) the number of decision trees (given the values 3, 10, 25 and 40 trees) and (2) the random seed number (used 5 random seeds).

## Results

### Testing the extract, transform and load procedure

**Figure 2:**
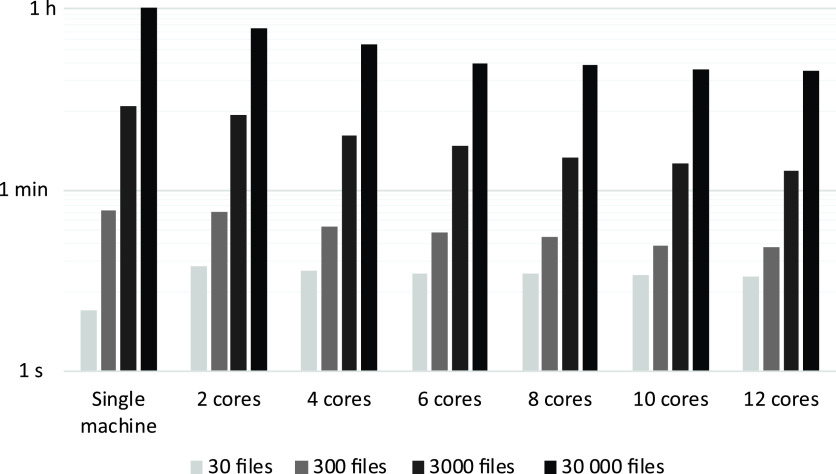
Wall time (s, min and h) for converting binary force plate data of turkeys into comma-separated file format and storing them on Hadoop Distributed File System (HDFS). The x-axis depicts the number of cores for each configuration, whereas the y-axis is the wall time (note the logarthimic scale).

Table 2 summarizes the number of turkeys and the total file sizes in binary and open formats for each of the four generated data sets. The required wall time (reported in a logarithmis scale) to transform these data sets into CSV files is summarized in Figure 2. Clearly, data sizes increase after the ETL-procedure has been applied and range from 18.6 MB for a data set containing data from 30 turkeys to 18.2 GB for a set containing data from 30 000 turkeys.

**Table 2:** File sizes in original binary format and after the ETL-procedure testing the turkey data preprocessing and data analyses

Number of turkeys	Original data size (binary format)	Data size after the ETL-procedure (open data format)
30	8.4 MB	18.6 MB
300	84 MB	186.1 MB
3000	837 MB	1.8 GB
30 000	8.4 GB	18.2 GB

ETL = extraxt, transform and load.

The required wall time (reported on a logarithmic scale) to transform these four artifically generated data sets into CSV files is summarized in Figure 2. Clearly, for the small data set of only 30 turkeys, the use of Apache Spark slows down the ETL-procedure. However, for the larger data sets (300, 3000 and 30 000 turkeys) use of the data lake and Apache Spark resulted in noticable reduced wall times, being up to 75% faster compared to a single machine. In the case of 30 000 turkeys, 8.4 GB of binary files was extracted into the lake (Table 1), transformed into CSV files and loaded in HDFS. The total size of the loaded files was 18.2 GB (Table 1), and the ETL-procedure required about 1 h on a single computer. A cluster of 12 cores, however, was able to perform the same procedure in less than 15 min.

Table 3 summarizes the AUROC and AUPRC values for 5-fold cross-validation for the whole parameter space. Averages and SD of the two metrics are reported for five repetitions with different seeds. These results show that the model developed was able to distinguish between the two classes (very bad gait score or otherwise). The best performing model reached an AUPRC value of 0.756 using 25 trees and seed #5.

**Table 3:** Area under the receiver operating curve (AUROC) and the area under the precisision-recall curve (AUPRC) for the random forest classification models using 5-fold cross-validation of turkey data

Number of trees	AUROC	AUPRC
3	0.754 (0.04)	0.688 (0.05)
10	0.752 (0.02)	0.682 (0.04)
25	0.8 (0.02)	0.718 (0.04)
40	0.783 (0.01)	0.704 (0.03)

Reported average values over five different seeds (SD in brackets).

## Discussion

In the animal sciences domain, a plethora of data is generated and will be generated in the (near) future. Consequently, challenges raise how to store, process and access these large volumes of data with different formats. Here, we explored a data lake in which data from an animal experiment were collected, ingested and subsequently transformed and analysed to investigate the benefit and caveats of a data lake. The main advantage of a data lake approach is that the entire ‘universe’ of data from an animal experiment can be captured and maintained in one location. This strengthens the findable, accessible, interoperable and reusable (**FAIR**) guiding principles for data (Wilkinson *et al.*, 2016). For example, no data loss occurs and the data are stored in their raw (native) format. The image, instructions and accompanying metadata can be uploaded to repository managers, for example, GitLab, GitHub, or SourceForge. These repositories bring (software) developers together and facilitate to discover, share and build software. Additionally, it makes the data also more findable and reusable on such repositories for other scientists. Often it is mandatory to attach metadata and ‘read me’ files to the software; this is also in line with the FAIR guiding principles and may contribute to the reusability of the data.

### Building and managing a data lake

Before starting to work with a data lake, the type of data one is working with an important aspect to keep in mind, that is, open, shared or closed data. This will bring different strategies of building and utilizing any data lake. In the current study, we built a dedicated customized data lake to work with private data (*n* = 84 turkeys), because of the sensitivity of the data. However, for educational purposes we also built a data lake with a subset of open data (*n* = 3 turkeys).

The data lake implementation required installing various software packages, as well as their dependencies, which were necessary to transform the original format of the sensor data into an open format which could be used in different statistical methods.

We designed and built a data lake for (sensor) data of an animal experiment. During this process, we encountered two other important aspects associated with deploying a ‘data lake approach’: (1) the possible skills gap and (2) the rate of change in hardware and software. The skills gap in people has already been identified (Gesing *et al.*, 2015; Connor *et al.*, 2016) and the ‘people’ aspect is often more pressing in organizations compared to the ‘processes and technology’ aspect (Gibert *et al.*, 2018). This includes skills around command-line code (i.e. Linux and/or Bash) and other programming languages (including R, Java, Scala and Python). Within the animal sciences domain, where to our best knowledge not much effort is (yet) admitted to data lake approaches, this skills gap was also observed (Gibert *et al.*, 2018). To adopt data lake approaches, investments in people and their skills are therefore necessary. Reducing skills gap already starts by translating and adopting today’s challenges in the data lake approach into, for example, education of MSc and PhD students or in animal experimentation. For the latter, it is expected that experimental data will become larger in volume and more heterogeneous. With increasing complexity, data lakes are better in handling heterogeneous data compared to data warehouses (Leary, 2014; Miloslavskaya and Tolstoy, 2016).

Moreover, for precision livestock farming where it is expected that sensor data will become widely available in the (near) future, ingestion and storage of large volumes of data can be performed real-time by a data lake. In the current study, the greatest challenges were (1) the data transformations, due to the non-standardized raw data from the different sensors, and (2) the metadata, that is, information and/or data that describe the characteristics of data. By generating customized scripts, we managed to transform the original format of the sensor data in an automatic way and process the data for further analyses. The generation of metadata of this animal experiment was dependent on the logistics during the day of the trial. For example, for each individual turkey the IMU(s) needed to be strapped on and the animal identifier (i.e. wing band) was written down manually in the computer system as metadata. Subsequently, the FP data were stored in a folder which was renamed to the animal identifier, this was also done for the video data. This was a suboptimal setting in terms of recording the metadata, nevertheless a rich metadata file was generated encompassing all the turkeys and their associated data, that is, FP, IMU, 3D-video and gait score data. These metadata are important to efficiently manage your data lake, as without effective metadata, some data may never surface again.

Integrated data lake management platforms do exist, which makes metadata creation and maintenance an integral part of the data lake processes. However, automation of these processes will be more challenging for animal experiments, because of their nature of testing specific hypotheses. For real-time monitoring and large-scale deployments, these integrated data lake management platforms would be a more suitable solution.

### Extract, load and transform procedure

In our data lake, we have used common scripting languages, such as Python and C++. In retrospect, we have devoted ample time to generate these scripts to translate the original binary data formats from the FP and IMU sensors to more common interoperable formats, such as comma- or tab-separated files. This is an important aspect, because in this way the pre-processed data are more easily transferred between different machine configurations, that is, interoperability. For instance, when these data need to be queried by another machine the ETL-procedure is more straightforward. In future endeavours, it is expected that more data will be generated and the ETL-procedure will become more prominent as well. Thus, scaling-up to store these data will become more important. Already many dedicated platforms, like Microsoft Azure, Amazon Web Services or Google Cloud Platform, are available. Another aspect is the granularity, that is, the communication overhead between the different processers. We observed that for small data sets the overhead can be a factor that slows down the ETL-procedure, in relative computing time. Whereas when working with large data sets, the relative computing time is not hindered by the overhead.

### Data analytics

Creating value from all the data ingested in a data lake is a research domain in itself, often called data science. Recently, a review has been published about Big Data ML in animal science (Morota *et al.*, 2018). Here, we want to emphasize that it is possible to automate the whole process in the data lake by building customized ETL-procedure and data analytics pipelines. Such pipelines may encompass different statistical methods supplemented with specific visualization tools. Here, we have shown that the ETL-procedure was scalable and the wall time was less with eight or more cores compared to a single core configuration. Open data format files were used to extract features to predict gait score by employing Apache Spark ML pipeline. The resulting output did not bear any biological relevance and was for the sole purpose of emphasizing on the scalability and flexibility of an ML pipeline. Note that the scalability of Apache Spark ML pipelines and hyperparameter tuning have already been investigated for domain-independent case studies (Zaharia *et al.*, 2010; Salloum *et al.*, 2016; Zaharia *et al.*, 2016). While this is not an exhaustive search of the parameter space for the particilar model, and several other approaches could have been used for both extracting features, learning from data and fine-tune the learning process, we demonstrated how ML pipelines can be used together with the ETL-procedure in a data lake environment for an animal experiment. This is an encouraging outcome for future research, taking into acount that the features used as model inputs were both highly noisy and lacked any biological meaning.

## Conclusions

In the era of Big Data, we expect animal science research will rely on information of high volume and uncertainty. This work demonstrated how to process sensor data using data lakes, with a scalable scientific workflow and ML models that perform with noisy data. We managed to build a data lake and ingest sensor data from an animal experiment, as well as subsequently to run an ML pipeline. We have also shown that it is possible to scale up the ETL-procedure, which may become more important when data need to be processed real-time for management (i.e. automated decision making). A data lake appears to be a useful tool to face the challenge of storing, combining and analysing increasing volumes of data of varying nature in an effective manner.

## Software and data repository resources

All developed scripts, Jupyter notebooks and the Docker container are available online through github, https://github.com/BigDataWUR/locomotiondatalake.git, using an open-source license. Only the educational data set, that is, three turkeys, is freely available through github or at zenodo; https://zenodo.org/record/3563513#.XsI0XmgzaUk.
